# Supplementary material for: Inappropriate Prescriptions in Older People—Translation and Adaptation to Portuguese of the STOPP/START Screening Tool
Source: Int J Environ Res Public Health. 2022 Jun 4;19(11):6896. doi: 10.3390/ijerph19116896 (PMC9180165; doi:10.3390/ijerph19116896)
Supplement: Supplementary file 1 [file ijerph-19-06896-s001.zip › IJERPH - Supplementary Material S1_Informed Consent.pdf]

CONSENTIMENTO INFORMADO, LIVRE E ESCLARECIDO

PARA PARTICIPAÇÃO EM INVESTIGAÇÃO

Título do estudo: critérios STOPP/START (versão 2) operacionalizados para Portugal

Enquadramento: Estudo observacional, em unidades de saúde em Portugal continental. Feito no âmbito de tese de doutoramento da Faculdade de Medicina da Universidade do Porto de Luís Monteiro, orientado pela Prof. Doutor Carlos Martins, Prof.ª Andreia Teixeira e Prof.ª Matilde Monteiro-Soares.

Explicação do estudo: Estudo observacional longitudinal efetuado após consulta médica, com aplicação dos critérios STOPP/START traduzidos previamente para português.

Estudo feito com pessoas com idade igual ou superior a 65 anos que recorrem a consulta, que aceitem participar no estudo e saibam ler. Serão recolhidas as variáveis: sexo, idade, formação (número de anos de escolaridade), índice socioeconómico, antecedentes pessoais, patologias atuais e no passado, inquirindo os utentes e verificando o registado nos processos clínicos.

Condições e financiamento: Não há pagamentos a investigadores ou participantes, nem compensação de despesas de deslocação. O estudo foi aprovado pela comissão de ética da ARS Centro. A participação no estudo é voluntária e caso não queira participar ou queira abandonar a qualquer altura, não será prejudicado.

Confidencialidade e anonimato: cada investigador terá uma base de identificação dos seus utentes, identificação esta codificada nos dados em Excel que serão enviados ao investigador principal. A identificação dos participantes nunca terá de ser tornada pública.

O investigador:

Assinatura: Data:

Declaro ter lido e compreendido este documento, bem como as informações verbais que me foram fornecidas pela/s pessoa/s que acima assina/m. Desta forma, aceito participar neste estudo e permito a utilização dos dados que de forma voluntária forneço, confiando que apenas serão utilizados para esta investigação e nas garantias de confidencialidade e anonimato que me são dadas.

Nome do utente:

Assinatura: Data:

Este documento composto de 1 página, é feito em duplicado, uma via para o investigador e outra para o utente.
