# Supplementary material for: Inappropriate Prescriptions in Older People—Translation and Adaptation to Portuguese of the STOPP/START Screening Tool
Source: Int J Environ Res Public Health. 2022 Jun 4;19(11):6896. doi: 10.3390/ijerph19116896 (PMC9180165; doi:10.3390/ijerph19116896)
Supplement: Supplementary file 1 [file ijerph-19-06896-s001.zip › IJERPH - Supplementary Material S2_STOPP.START Translation.pdf]

International Journal of
Environmental Research and Public Health

MDPI

Supplementary Material S2

Inappropriate prescriptions in older people - Translation and adaptation to Portuguese of the STOPP/START screening tool

Luís Monteiro, Matilde Monteiro-Soares, Cristiano Matos, Inês Ribeiro-Vaz, Andreia Teixeira and Carlos Martins

Correspondence: luismonteiro.net@gmail.com (L.M.)

Translation and adaptation from: [20] - O'Mahony D et al. STOPP/START criteria for potentially inappropriate prescribing in older people: version 2. Age and Ageing. 2014;44(2):213-8.

Screening Tool of Older Persons' Prescriptions (STOPP) (Ferramenta de avaliação da prescrição de idosos) – versão 2 (2014).

As seguintes prescrições são potencialmente inadequadas para administração em doentes com 65 anos ou mais.

Secção A: Indicação de medicação

1. Qualquer medicamento prescrito sem indicação clínica baseada em evidência.
2. Qualquer medicamento prescrito para além da duração recomendada, sempre que a duração do tratamento estiver bem definida.
3. Qualquer prescrição de classe de medicamentos em duplicado, por exemplo, dois AINE (anti-inflamatórios não esteroides), (ISRS) inibidores seletivos da recaptação da serotonina (ISRS), diuréticos da ansa, inibidores da ECA (enzima conversora da angiotensina), anticoagulantes, em simultâneo (a otimização da monoterapia com uma única classe de medicamentos deve ser observada antes de considerar um novo agente).

Secção B: Sistema cardiovascular

1. Digoxina para insuficiência cardíaca com função ventricular sistólica normal (sem evidência clara de benefícios).
2. Verapamil ou diltiazem na insuficiência cardíaca classe III ou IV da *New York Heart Association* (NYHA) (pode agravar a insuficiência cardíaca).
3. Bloqueadores beta em combinação com verapamil ou diltiazem (risco de bloqueio cardíaco).
4. Bloqueador beta com bradicardia (<50/min), bloqueio auriculoventricular de segundo grau ou bloqueio auriculoventricular completo (risco de bloqueio atrioventricular completo, assistolia).
5. Amiodarona como terapêutica antiarrítmica de primeira linha em taquiarritmias supraventriculares (risco mais elevado de efeitos secundários do que bloqueadores beta, digoxina, verapamil ou diltiazem).
6. Diuréticos da ansa como tratamento de primeira linha para hipertensão (alternativas disponíveis mais seguras e mais eficazes).
7. Diuréticos da ansa para edema maleolar sem evidência clínica, bioquímica ou radiológica de insuficiência cardíaca, insuficiência hepática, síndrome nefrótica ou insuficiência renal (elevação das pernas e / ou compressão elástica degressiva, normalmente, mais adequadas).
8. Diuréticos tiazídicos na presença de hipocalemia significativa (ou seja, potássio (K+) sérico <3,0 mmol/l), hiponatremia (ou seja, sódio (Na+) sérico <130 mmol/l), hipercalcemia (ou seja, cálcio sérico corrigido >2,65 mmol/l) ou com antecedentes de gota (hipocalemia, hiponatremia, hipercalcemia e gota podem ser desencadeados por diuréticos tiazídicos).
9. Diuréticos da Ansa para tratamento da hipertensão na presença de incontinência urinária (pode exacerbar a incontinência).
10. Anti-hipertensores de ação central (por exemplo: metildopa, clonidina, moxonidina, rilmenidina, guanfacina), exceto em caso de intolerância clara a, ou falta de eficácia de, outras classes de anti-hipertensores (os anti-hipertensores de ação central são, geralmente, menos bem tolerados por pessoas mais idosas do que por pessoas mais jovens).

11. Inibidores da ECA (enzima conversora da angiotensina) ou bloqueadores do recetor da angiotensina em doentes com hipercalemia.
12. Antagonistas da aldosterona (por exemplo, espironolactona, epleronona) em simultâneo com medicamentos preservadores do potássio [por exemplo, IECA (inibidores da enzima conversora da angiotensina), ARB (bloqueadores do recetor da angiotensina), amilorida, triantereno)] sem monitorização do potássio sérico (risco de hipercalemia grave, ou seja, >6,0 mmol/l – o potássio sérico deve ser monitorizado regularmente, ou seja, pelo menos, de 6 em 6 meses).
13. Inibidores da fosfodiesterase 5 (por exemplo, sildenafil, taladafil, vardenafil) na insuficiência cardíaca grave caracterizada por hipotensão, ou seja, pressão arterial sistólica <90 mmHg, ou terapêutica conjunta com nitratos para angina de peito (risco de colapso cardiovascular).

Secção C: Medicamentos antiagregantes/anticoagulantes

1. Tratamento a longo prazo com ácido acetilsalicílico em doses superiores a 160 mg por dia (risco aumentado de hemorragia, sem evidência de aumento da eficácia).
2. Ácido acetilsalicílico com antecedentes de doença ulcerosa péptica sem terapêutica concomitante com IBP (inibidores da bomba de protões) (risco de recidiva de doença ulcerosa péptica).
3. Ácido acetilsalicílico, clopidogrel, dipiridamol, antagonistas da vitamina K, inibidores diretos da trombina ou inibidores do fator Xa com presença significativa de risco de hemorragia, ou seja, hipertensão grave não controlada, diátese hemorrágica, hemorragia espontânea recente não habitual (risco elevado de hemorragia).
4. Ácido acetilsalicílico mais clopidogrel como prevenção de AVC (Acidente Vascular Cerebral) secundário, a menos que o doente tenha um *stent* ou *stents* coronário(s) introduzido(s) nos 12 meses anteriores ou simultânea síndrome coronária aguda, ou tenha um elevado grau de estenose carotídea sintomática (não há evidência de benefício acrescentado à monoterapia com clopidogrel).
5. Ácido acetilsalicílico em combinação com antagonistas da vitamina K, inibidores diretos da trombina ou inibidores do fator Xa em doentes com fibrilhação auricular crónica (sem benefício acrescentado com o ácido acetilsalicílico).
6. Agentes antiagregantes com antagonistas da vitamina K, inibidores diretos da trombina ou inibidores do fator Xa em doentes com doença coronária, cerebrovascular ou arterial periférica estável (sem benefício acrescentado com terapêutica dupla).
7. Ticlopidina em quaisquer circunstâncias (clopidogrel e prasugrel têm eficácia similar, evidência mais sólida e menos efeitos secundários).
8. Antagonistas da vitamina K, inibidores diretos da trombina ou inibidores do fator Xa para primeira trombose venosa profunda sem fatores de risco permanentes (por exemplo, trombofilia) durante > 6 meses (sem acréscimo comprovado de benefícios).
9. Antagonistas da vitamina K, inibidores diretos da trombina ou inibidores do fator Xa para primeira embolia pulmonar sem fatores de risco permanentes (por exemplo, trombofilia) durante > 12 meses (sem acréscimo comprovado de benefícios).
10. AINE em combinação com antagonistas da vitamina K, inibidores diretos da trombina ou inibidores do fator Xa (risco de grande hemorragia gastrointestinal).
11. AINE conjuntamente com agente(s) antiplaquetário(s) sem profilaxia com IBP (risco aumentado de doença ulcerosa péptica).

Secção D: Sistema nervoso central e medicamentos psicotrópicos

1. Antidepressivos tricíclicos (ADT) com demência, glaucoma de ângulo fechado, anomalias da condução cardíaca, prostatismo ou antecedentes de retenção urinária (risco de agravar estas condições).
2. Início de ADT como tratamento antidepressivo de primeira linha (risco mais elevado de reações adversas ao medicamento com ADT do que com inibidores seletivos da recaptação da serotonina (ISRS) ou inibidores seletivos da recaptação da serotonina e da noradrenalina (ISRSN).
3. Neurolépticos com efeitos moderadamente marcados antimuscarínicos/anticolinérgicos (clorpromazina, clozapina, flupentixol, flufenazina, pipotiazina, promazina, zuclopentixol) com antecedentes de prostatismo ou retenção urinária (risco elevado de retenção urinária).
4. Inibidores seletivos da recaptação da serotonina (ISRS) com atual ou recente hiponatremia significativa, ou seja, Na+ sérico < 130 mmol/L (risco de exacerbar ou desencadear hiponatremia).

5. Benzodiazepinas durante ≥ 4 semanas (sem indicação para tratamento mais longo; risco de sedação prolongada, confusão, dificuldades de equilíbrio, quedas, acidentes de viação; todas as benzodiazepinas devem ser retiradas gradualmente se tomadas por mais de 4 semanas, porque existe o risco de provocar a síndrome de abstinência de benzodiazepinas se a administração das mesmas for interrompida abruptamente).
6. Antipsicóticos (ou seja, outros além da quetiapina ou clozapina) em doentes com parkinsonismo ou demência por corpos de *Lewy* (risco de sintomas extrapiramidais graves)).
7. Anticolinérgicos/antimuscarínicos para tratar efeitos secundários extrapiramidais de medicação neuroléptica (risco de toxicidade anticolinérgica).
8. Anticolinérgicos/antimuscarínicos em doentes com *delirium* ou demência (risco de exacerbar dificuldades cognitivas).
9. Antipsicótico neuroléptico em doentes com sintomas psicológicos e comportamentais da demência (SPCD), a menos que os sintomas sejam graves e outros tratamentos não farmacológicos tenham falhado (risco aumentado de AVC).
10. Neurolépticos como hipnóticos, a menos que os distúrbios do sono sejam devidos a psicose ou demência (risco de confusão, hipotensão, efeitos secundários extrapiramidais, quedas).
11. Inibidores da acetilcolinesterase com antecedentes conhecidos de bradicardia persistente (<60 batimentos/min), bloqueio cardíaco, ou síncope inexplicada, ou tratamento conjunto com medicamentos que reduzem o ritmo cardíaco, tais como betabloqueadores, digoxina, diltiazem, verapamil (risco de falha da condução cardíaca, síncope e lesão).
12. Fenotiazinas como tratamento de primeira linha, dado que existem alternativas mais seguras e mais eficazes (as fenotiazinas têm efeito sedativo, têm uma toxicidade antimuscarínica significativa em pessoas idosas, à exceção de proclorperazina para náuseas/vómitos/vertigens, clorpromazina para alívio de soluços persistentes e levomepromazina como antiemético em cuidados paliativos).
13. Agonistas da levodopa ou da dopamina para tremor essencial benigno (sem evidência de eficácia).
14. Anti-histamínicos de primeira geração (há atualmente outros anti-histamínicos mais seguros, menos tóxicos e amplamente disponíveis).

Secção E: Sistema renal. Os medicamentos seguintes são potencialmente inadequados em pessoas idosas com doença renal aguda ou crónica, com função renal abaixo dos níveis específicos da taxa de filtração glomerular estimada (TFGe) (consulte as tabelas do resumo das características do produto e as orientações do formulário local)

1. A digoxina numa dose a longo prazo superior a 125µg/dia e TFGe <30 ml/min/1,73m2 (risco de toxicidade da digoxina se os níveis plasmáticos não forem aferidos).
2. Inibidores diretos da trombina (por exemplo, dabigatrano) se TFGe <30 ml/min/1,73m2 (risco de hemorragia).
3. Inibidores do fator Xa (por exemplo, rivaroxabano, apixabano) se TFGe <15 ml/min/1,73m2 (risco de hemorragia).
4. AINE se TFGe <50 ml/min/1,73m2 (risco de deterioração da função renal).
5. Colquicina se TFGe <10 ml/min/1,73m2 (risco de toxicidade da colquicina).
6. Metformina se TFGe <30 ml/min/1,73m2 (risco de acidose láctica).

Secção F: Sistema gastrointestinal

1. Proclorperazina ou metoclopramida com parkinsonismo (risco de exacerbar os sintomas de parkinsonismo).
2. IBP para doença ulcerosa péptica não complicada ou esofagite péptica erosiva e dosagem terapêutica completa durante > 8 semanas (indicação para redução da dose ou descontinuação do tratamento mais cedo).
3. Medicamentos passíveis de provocar obstipação (por exemplo, medicamentos antimuscarínicos/anticolinérgicos, ferro por administração oral, opioides, verapamil, antiácidos à base de alumínio) em doentes com obstipação crónica quando estiverem disponíveis alternativas que não causem obstipação (risco de exacerbar a obstipação).

4. Doses de ferro elementar por administração oral superiores a 200 mg por dia (por exemplo, fumarato ferroso >600 mg/dia, sulfato ferroso >600 mg/dia, gluconato ferroso >1800 mg/dia; sem evidência de melhoria da absorção de ferro acima destas doses).

Secção G: Sistema respiratório

1. Teofilina como monoterapia para DPOC (doença pulmonar obstrutiva crónica) (alternativa mais segura, mais eficaz; risco de efeitos secundários devido a margem terapêutica estreita).
2. Corticosteróides sistémicos em vez de corticosteróides inalados para terapêutica de manutenção em DPOC moderada ou severa (exposição desnecessária a efeitos secundários sistémicos de corticosteróides a longo prazo e estão disponíveis terapêuticas inalatórias eficazes).
3. Broncodilatadores antimuscarínicos (por exemplo, ipratróprio, tiotróprio) com antecedentes de glaucoma de ângulo fechado (pode exacerbar o glaucoma) ou obstrução do fluxo urinário (pode causar retenção urinária).
4. Bloqueador beta não seletivo (quer por via oral quer por aplicação tópica para glaucoma) com antecedentes de asma com necessidade de tratamento (risco de aumentar broncoespasmos).
5. Benzodiazepinas com insuficiência respiratória aguda ou crónica, ou seja, pO2 <8,0 kPa ou <60 mmHg ± pCO2 >6,5 kPa ou >48,8 mmHg (risco de exacerbar a insuficiência respiratória).

Secção H: Sistema musculoesquelético

1. AINE que não seja agente seletivo da COX-2, com história de doença ulcerosa péptica ou hemorragia gastrointestinal, exceto com a administração simultânea de IBP ou antagonistas dos recetores H2 (risco de recidiva de úlcera péptica).
2. AINE com hipertensão grave (risco de exacerbar a hipertensão) ou insuficiência cardíaca grave (risco de exacerbar a insuficiência cardíaca).
3. AINE a longo prazo (>3 meses) para alívio dos sintomas de dor causada por osteoartrose, quando não se tiver tentado o paracetamol (analgésico simples, preferível e, normalmente, mais eficaz para alívio da dor).
4. Corticosteróides a longo prazo (>3 meses) em monoterapia para artrite reumatóide (risco de efeitos secundários sistémicos de corticosteroides).
5. Corticosteróides (outros que não sejam injeções intra-articulares periódicas para dor em apenas uma articulação) para osteoartrose (risco de efeitos secundários sistémicos de corticosteroides).
6. AINE a longo prazo ou colquicina (>3 meses) para tratamento crónico da gota, quando não houver contraindicações para um inibidor da xantina oxidase (o alopurinol ou febuxostate, dado que os inibidores da xantina oxidase são fármacos de primeira linha na profilaxia da crise aguda de gota).
7. AINE seletivos da COX-2 com doença cardiovascular concomitante (risco aumentado de enfarte do miocárdio e AVC).
8. AINE combinados com corticosteróides sem profilaxia com IBP (risco aumentado de doença ulcerosa péptica).
9. Bifosfonatos por administração oral em doentes com antecedentes de doença no trato gastrointestinal superior, ou seja, disfagia, esofagite, gastrite, duodenite, ou doença ulcerosa péptica, ou hemorragia do trato gastrointestinal superior (risco de recidiva/exacerbação de esofagite, úlcera esofágica, estenose do esófago).

Secção I: Sistema urogenital

1. Medicamentos antimuscarínicos na demência, ou dificuldades cognitivas crónicas (risco de aumento de confusão, agitação), ou glaucoma do ângulo fechado (risco de exacerbação aguda do glaucoma), ou prostatismo crónico (risco de retenção urinária).
2. bloqueadores alfa-1 seletivos em doentes com hipotensão ortostática sintomática ou síncope durante a micção (risco de provocar síncope recorrente).

Secção J. Sistema endócrino

1. Sulfonilureias com ação de longa duração (por exemplo, glibenclamida, clorpropamida, glimepirida) na diabetes *mellitus* tipo 2 (risco de hipoglicemia prolongada).
2. Tiazolidinedionas por exemplo, rosiglitazona, pioglitazona) em doentes com insuficiência cardíaca (risco de exacerbar a insuficiência cardíaca).

3. Bloqueadores beta na diabetes *mellitus* com episódios hipoglicémicos frequentes (risco de supressão dos sintomas hipoglicémicos).
4. Estrogénios com história de cancro da mama ou tromboembolismo venoso (risco aumentado de recidivas).
5. Estrogénios orais sem progesterona em doentes com o útero intacto (risco de cancro do endométrio).
6. Androgénios (hormonas do sexo masculino) na ausência de hipogonadismo primário ou secundário (risco de toxicidade por androgénios; não existem benefícios comprovados para além da indicação para hipogonadismo).

Secção K: Medicamentos que aumentam, previsivelmente, o risco de quedas em pessoas idosas

1. Benzodiazepinas (efeito sedativo, podem causar redução dos sentidos, dificuldades de equilíbrio).
2. Medicamentos neurolépticos (podem causar dispraxia durante a marcha, parkinsonismo).
3. Medicamentos vasodilatadores (por exemplo, bloqueadores dos recetores alfa-1, bloqueadores dos canais de cálcio, nitratos de longa duração de ação, inibidores da ECA, bloqueadores do recetor I da angiotensina) com hipotensão postural persistente, ou seja, queda frequente da pressão arterial sistólica ≥20mmHg (risco de síncope, quedas).
4. Medicamentos Z-hipnóticos, por exemplo, zopiclona, zolpidem, zaleplon (podem causar sedação prolongada durante o dia, ataxia).

Secção L: Medicamentos analgésicos

1. Utilização de opioides fortes transdérmicos (morfina, oxicodona, fentanilo, buprenorfina, diamorfina, metadona, tramadol, petidina, pentazocina) como terapêutica de primeira linha para dor ligeira (escada analgésica da Organização Mundial de Saúde não observada).
2. Utilização regular (distinto de quando necessário) de opióides sem fármacos laxantes em simultâneo (risco de obstipação grave).
3. Opióides de longa ação sem opióides de curta ação para combater a dor (risco de persistência de dor intensa).

Secção N: Carga medicamentosa antimuscarínica/anticolinérgica

Utilização concomitante de dois ou mais medicamentos com propriedades antimuscarínicas/ anticolinérgicas (por exemplo, antiespasmódicos das vias urinárias, antiespasmódicos intestinais, antidepressivos tricíclicos, anti-histamínicos de primeira geração (risco aumentado de toxicidade antimuscarínica/ anticolinérgica).

Screening Tool to Alert to Right Treatment (START), (Ferramenta de avaliação para alertar para o tratamento correcto) versão 2 (2014)

A menos que o estado clínico de um doente idoso seja terminal, requerendo, assim, uma abordagem farmacoterapêutica mais paliativa, as seguintes terapêuticas farmacológicas devem ser consideradas, quando não foram prescritas sem fundamento(s) clínico(s) válido(s). Presume-se que quem faz a prescrição observe todas as contraindicações específicas destas terapêuticas farmacológicas antes de as recomendar a doentes idosos.

Secção A: Sistema cardiovascular

1. Antagonistas da vitamina K, ou inibidores diretos da trombina, ou inibidores do fator Xa na presença de fibrilhação auricular crónica.
2. Ácido acetilsalicílico (75 mg - 160 mg, uma vez por dia) na presença de fibrilhação auricular crónica, quando estiverem contraindicados antagonistas da vitamina K, ou inibidores diretos da trombina ou inibidores do fator Xa.
3. Terapêutica antiagregante (Ácido acetilsalicílico, ou clopidogrel, ou prasugrel, ou ticagrelor) com antecedente de doença coronária, cerebral ou vascular periférica.
4. Terapêutica anti-hipertensora quando a pressão arterial sistólica for consistentemente >160 mmHg e/ou a pressão arterial diastólica for consistentemente >90 mmHg; se a pressão arterial sistólica for >140 mmHg e/ou a pressão arterial diastólica for >90 mmHg, em doente diabético.
5. Terapêutica com Estatinas com um historial documentado de doença coronária, cerebral ou vascular periférica, a menos que o estado do doente seja terminal ou com idade >85 anos.
6. Inibidores da enzima conversora da angiotensina (ECA) na insuficiência cardíaca sistólica e/ou doença arterial coronária documentada.
7. Bloqueador beta na doença cardíaca isquémica.
8. Bloqueador beta adequado (bisoprolol, nebivolol, metoprolol ou carvedilol) na insuficiência cardíaca sistólica estável.

Secção B: Sistema respiratório

1. Agonistas β2 inalados ou broncodilatadores antimuscarínicos usuais (por exemplo, ipratróprio, tiotróprio) para asma ligeira a moderada ou DPOC.
2. Corticosteróides inalados usuais para asma moderada a grave ou DPOC, quando FEV1 <50 % do valor previsto e ocorrerem exacerbações repetidas que exijam tratamento com corticosteróides orais.
3. Oxigénio de longa duração no domicílio com hipoxemia crónica documentada (ou seja, pO2 <8,0 kPa ou 60 mmHg ou SaO2 <89%).

Secção C: Sistema nervoso central e olhos

1. Levodopa ou um agonista da dopamina na doença de Parkinson idiopática com perturbações funcionais e consequente incapacidade.
2. Medicamento antidepressivo não ADT na presença de sintomas depressivos graves persistentes.
3. Inibidor da acetilcolinesterase (por exemplo, donepezilo, rivastigmina, galantamina) para demência de Alzheimer ligeira a moderada ou demência por corpos de Lewy (rivastigmina).
4. Prostaglandinas tópicas, prostamidas ou bloqueadores beta para glaucoma primário de ângulo aberto.
5. Inibidores seletivos da recaptação da serotonina (ou Inibidores seletivos da recaptação da serotonina e da noradrenalina (ISRSN) ou pregabalina, se ISRS estiverem contraindicados) para ansiedade grave persistente que interfira com o funcionamento independente.
6. Agonista da dopamina (ropinirol, ou pramipexol, ou rotigotina) para síndrome das pernas inquietas, quando estiverem excluídas a deficiência de ferro e a insuficiência renal grave.

Secção D: Sistema gastrointestinal

1. Inibidores da bomba de protões com doença de refluxo gastroesofágico grave ou estenose péptica que exija dilatação.
2. Suplementos de fibra (ou seja, farelos, ispágula, metilcelulose, estercúlia) para diverticulose com historial de obstipação.

Secção E: Sistema musculoesquelético

1. Medicamento antirreumático modificador da doença (MARMD) com doença reumática ativa e incapacitante.
2. Bifosfonatos, vitamina D e cálcio em doentes a tomar medicação corticosteroide sistémica a longo prazo.
3. Suplementos de vitamina D e cálcio em doentes com osteoporose conhecida e/ou fratura(s) de fragilidade anterior e/ou (índice T da densidade mineral óssea superior a -2,5 em vários locais).
4. Terapêutica anti-reabsortiva óssea ou anabolizante (por exemplo, bifosfonato, ranelato de estrôncio, teriparatida, denosumab) em doentes com osteoporose documentada, quando não existir qualquer contraindicação farmacológica ou do estado clínico (índice T da densidade mineral óssea - >2,5 em vários locais) e/ou historial anterior de fratura(s) de fragilidade.
5. Suplementos de vitamina D em pessoas idosas que estejam confinadas em casa ou que tenham sofrido quedas ou com osteopenia (índice T da densidade mineral óssea é >-1,0, porém <-2,5 em vários locais).
6. Inibidores da xantina oxidase (por exemplo, alopurinol, febuxostate) com antecedentes de episódios de gota recorrentes.
7. Suplementos de ácido fólico em doentes a tomar metotrexato.

Secção F: Sistema endócrino

1. Inibidor da ECA ou bloqueador do recetor da angiotensina (se for intolerante a inibidor da ECA) na diabetes com evidência de doença renal, ou seja, tira reagente positiva para proteinúria ou microalbuminúria (>30 mg/24 horas) marcadores bioquímicos séricos de insuficiência renal.

Secção G: Sistema urogenital

1. Bloqueador do recetor alfa-1 com prostatismo sintomático, quando a prostatectomia não for considerada necessária.
2. Inibidores da 5-alfa-redutase com prostatismo sintomático, quando a prostatectomia não for considerada necessária.
3. Estrogénio vaginal tópico ou estrogénio em óvulo vaginal para atrofia vaginal sintomática.

Secção H: Analgésicos

1. Opióides de elevada potência em dor moderada a intensa, quando o paracetamol, AINE ou opióides de baixa potência não forem adequados para a intensidade da dor ou não foram eficazes.
2. Laxantes em doentes que tomam opióides regularmente.

Secção I: Vacinas

1. Vacina tetravalente contra a gripe sazonal todos os anos.
2. Vacina pneumocócica pelo menos uma vez após os 65 anos, de acordo com as orientações nacionais.

References

20. O'Mahony, D.; O'Sullivan, D.; Byrne, S.; O'Connor, M.N.; Ryan, C.; Gallagher, P. STOPP/START criteria for potentially inappropriate prescribing in older people: Version 2. *Age Ageing* **2014**, *44*, 213–218.
